# Learning how to conduct medical interviews online for the first time – this is what we learned in Frankfurt am Main

**DOI:** 10.3205/zma001415

**Published:** 2021-01-28

**Authors:** Judith Ullmann-Moskovits, Maria Farquharson, Miriam Schwär, Monika Sennekamp

**Affiliations:** 1 Goethe-Universität Frankfurt am Main, Institut für Allgemeinmedizin, Frankfurt/Main, Germany

**Keywords:** blended learning, history taking, communication, conversation skills, online teaching, digital didactics, medical education

## Abstract

**Objective: **The COVID-19 pandemic made it necessary to convert a course on history taking, in theory and practice, to an online format over a very short time. A key question was whether, and if so to what extent, basic theory and, in particular, the practical skills required to conduct medical interviews can be learned online.

**Methodology/project description: **The teaching program in basic theory was didactically redesigned and asynchronously placed on a learning platform, while the practical program, which consisted of training in conducting history-taking interviews, took place with the help of video conferencing software during synchronous sessions. For the practical sessions, the lecturers received organizational and technical support.

**Results:** Based on initial evaluation results, a positive picture of the conversion has emerged since the course was completed. The need to restructure the course and use new teaching methods because of the COVID-19 pandemic was well accepted by lecturers and students, and the course content was successfully adapted to an online format.

**Conclusion: **Overall, the online format enabled the learning objectives of the course to be successfully achieved. For topics such as non-verbal communication, the evaluation results indicated that a classroom format is preferable. Asynchronous theory teaching was generally very well received. Blended learning formats thus represent an appropriate means of teaching how to conduct medical interviews. Overall, online courses on conducting medical interviews provide students with the opportunity to become acquainted with the use of digital formats to conduct doctor-patient interviews, and to develop the relevant skills.

## Zusammenfassung

**Zielsetzung: **Aufgrund der COVID-19-Pandemie musste ein Anamnesekurs, bestehend aus Theorie und Praxis, innerhalb kürzester Zeit auf ein Onlineformat umgestellt werden. Eine zentrale Frage war, ob bzw. inwiefern erste theoretische und insbesondere praktische Grundlagen für die ärztliche Gesprächsführung online erlernbar sind.

**Methodik/Projektbeschreibung: **Die theoretischen Grundlagen wurden asynchron auf einer Lernplattform eingestellt sowie didaktisch neu konzipiert, während der Praxisteil zur Einübung der Anamnesegespräche in synchronen Treffen mithilfe von Videokonferenzsoftware stattfand. Die Dozierenden des Praxisteils wurden organisatorisch und technisch unterstützt.

**Ergebnisse: **Nach Abschluss des Kurses und basierend auf ersten Evaluationsergebnissen zeichnet sich ein positives Bild der Umstellung ab: Dozierende und Studierende nahmen die Umstrukturierung des Kurses aufgrund der COVID-19-Pandemie gut an und durch aktivierende didaktische Methoden ist es gelungen, die Inhalte des Kurses erfolgreich an das Onlineformat anzupassen.

**Schlussfolgerung: **Die Lernziele des Kurses konnten durch das Onlineformat insgesamt gut erreicht werden. Bei Themen, wie z.B. der nonverbalen Kommunikation erscheint anhand der Evaluationsergebnisse ein Präsenzformat empfehlenswert. Die asynchrone Theorievermittlung wurde insgesamt sehr gut angenommen. Blended-Learning-Formate stellen also eine adäquate Option für die Vermittlung ärztlicher Gesprächsführung dar. Insgesamt bieten Onlinekurse zur Gesprächsführung eine Möglichkeit, Studierende für digitale Formate der Arzt-Patienten-Gesprächsführung zu sensibilisieren und diesbezügliche Kompetenzen zu fördern.

## 1. Introduction

As a result of the COVID-19 pandemic, the Institute of General Practice at Goethe University Frankfurt was faced with the challenge of quickly converting the Introduction to Clinical Medicine (EKM) course to a new, digitally supported teaching-learning format for theoretical and practical teaching, and practicing the basics of conducting medical history interviews. The aim of this field report is to share our didactic considerations and findings.

The EKM course takes place every summer semester and is mandatory for all 400 medical students in their fourth pre-clinical semester. Postponing the course to the winter semester was ruled out for organizational reasons. The original course consists of seven two-hour sessions during which students learn basic theory and practice conducting medical history interviews. By the end of the course, all students should have independently conducted at least one medical history interview with a patient using the theoretical knowledge they have acquired. Trained lecturers from various clinical departments hold the 28 parallel courses, each of which is held with a group of 14 students. The medical history interviews take place with real and simulated patients. 

During the COVID-19 pandemic and in line with university requirements promoting the use of asynchronous teaching-learning methods, a blended learning format was used. Theory and practice were separated from one another [1], while the original learning objectives were maintained. For didactic reasons (first practical communication course in the curriculum), it was important to us to conduct the history taking interviews in a synchronous format.

## 2. Project description

### The asynchronous theory modules

The theoretical basics were processed asynchronously, module by module, and made available for self-study on the OLAT learning platform. The content included such topics as the structure of a medical history interview, questioning techniques, communication theories, etc. Time constraints made it necessary to develop the modules and put them online weekly. Diagrams, videos, case studies, exercises, and impulses for personal reflection, were added to the existing course script in order to do justice to the online format from a didactic perspective [2]. The common thread running through the course consisted of learning objectives, take home messages and instructions for individual practice, along with clear deadlines as structuring elements. 

#### The synchronous practice modules

Each group participated in four two-hour sessions during which the students conducted 3-4 medical history interviews with (simulated) patients. Various video conferencing systems were used depending on the technical equipment available. To help structure the teaching program, the lecturers were provided with an Excel list of the medical history interviews to be conducted per session. This file was also used to pre-structure the provision of mutual feedback by the students (a core element of the course) [3]. Based on the asynchronous learning units, the content of the feedback became increasingly thorough (e.g. questioning techniques, active listening). The new online format required specific didactic approaches, e.g. regular activation of the group using interactive methods such as breakout rooms or surveys to maintain students’ ability to concentrate. 

## 3. Results and discussion

Overall, it was possible to convey the basics of conducting medical interviews well in an online format. Based on the initial results of an evaluation adapted to the new format, we would like to summarize the most important lessons we learned and support those that are currently redesigning courses, or will be in the future:

- Lecturers and (simulated) patients must be prepared for and trained in using the online format in terms of content, didactics and technology (technology test, introduction to media usage, and new course structure). The resulting increase in the need to pre-structure the entire course is essential for its successful implementation.
- Data protection regulations must be adhered to and hospital guidelines on permitted video-conferencing systems observed, especially in a clinical context. For the theory modules, it is important that students have a clear overview of what they have to do, including clearly visible deadlines for tasks to be completed online, submission addresses and pass criteria.
- The structure requires a high degree of independence from students. Overall, they rated this aspect positively. In individual cases, however, multiple reminders were necessary.
- Asynchronous theory acquisition can relieve the work burden on lecturers and permit them to focus on teaching practical skills. During the synchronous practice modules, it was considered useful that time was available for reviewing theoretical aspects and getting questions answered.
- To a certain extent, it was also possible to establish personal contact online. However, activating a group dynamic that was conducive to learning success, and the development of a culture of discussion, was didactically challenging for lecturers in their role as moderators.
- It should be noted that online discussions differ from face-to-face discussions because there are fewer opportunities for non-verbal communication and there are technical limitations. The setting also creates a certain artificiality, which is something that was commented on in the online evaluation (questionnaire).
- In order to encourage constructive feedback, it is helpful to train lecturers, students and patients, and to allow sufficient time in the training sessions to provide feedback on previous feedback.
- Regarding the attention span, 120 minutes was stated as the maximum duration of the synchronous sessions.
- In the evaluations, students subjectively reported substantial learning progress.

## 4. Conclusion

Overall, the course change was positively accepted by all participants and has led to a significant increase in learning. Online and blended learning formats are an appropriate means of learning how to conduct medical interviews, as long as technical and didactic differences are taken into account. Given the choice, however, and based on the evaluation results, we believe that face-to-face sessions are more suitable for conducting medical history interviews. The online course on this topic provides the additional opportunity to discuss digital doctor-patient communication formats (e.g. telemedicine) and to promote the corresponding skills at an early stage.

## Competing interests

The authors declare that they have no competing interests. 
